# Multifocal cutaneous angiosarcoma of the scalp—A challenging reconstructive case managed with skin substitutes

**DOI:** 10.1002/cnr2.1659

**Published:** 2022-07-12

**Authors:** Henrique Messias, Mariluz Martins, Carlos Zagalo, Pedro Gomes

**Affiliations:** ^1^ Head and Neck Surgery Department Portuguese Institute of Oncology Francisco Gentil Lisbon Portugal; ^2^ Division of Health Sciences University of Edinburgh Edinburgh United Kingdom

**Keywords:** sarcoma, cutaneous angiossarcoma, head and neck, scalp, dermal substitute

## Abstract

**Background:**

Cutaneous angiosarcoma (AS) of the head and neck is a rare highly aggressive tumor, often associated with difficult local control of the disease and poor prognosis. This article describes a case of multifocal cutaneous AS of the scalp, mainly addressing its difficult surgical management and challenging reconstruction and concludes with a review of the literature.

**Methods:**

A 70‐year‐old Caucasian male was referred to our hospital with a growing scalp lesion initially suspected to be benign, but later diagnosed with AS.

**Results:**

The patient had tumor recurrence and a difficult reconstruction for which dermal substitutes proved very useful.

**Conclusion:**

AS can mimic a benign lesion in its early stages. Skin substitutes, namely dermal templates, can be useful to meet the complex needs of reconstruction and oncological surveillance of patients with AS.

**Abbreviations**
AS: angiosarcoma
AJCC: american joint committee on cancer
CAS: cutaneous angiosarcoma
CT: computerized tomography
DRT: dermal regeneration templates
END: elective lymph node dissection
FNAC: fine needle aspiration cytology
FNCLCC: fédération nationale des centres de lutte contre le cancer
HNCAS: head and neck cutaneous angiosarcoma
HNSA: head and neck sarcomas
LNM: lymph node metastasis
MDT: multidisciplinary head and neck oncology team
NPWT: negative pressure wound therapy
OS: overall survival
PET: positron emission tomography
PFS: progression‐free survival
VEGF: vascular endothelial growth factor

## 1 INTRODUCTION

Angiosarcoma (AS) is a rare, aggressive, malignant tumor, originating from vascular endothelial cells that can arise at any anatomical location.^1^, ^2^, ^3^, ^4^, ^5^ It accounts for less than 2% of all soft tissue sarcomas and less than 1% of head and neck cancers.^1^, ^3^ Cutaneous ASs are more common in the face and scalp, and, in these regions, most are sporadic.^4^ Incidence is increasing.^5^ Scalp AS is associated with challenging treatment and poor prognosis.^6^ Early detection is thus essential, with associated 10‐year survival rates reported as high as 53.6% in localized sarcomas, but as low as 13.8% in metastatic disease.^7^

This article describes a case of multifocal cutaneous AS of the scalp, reporting not only the early delay in the definitive diagnosis, but also the difficult surgical management secondary to its large defect and anatomic location, that was managed with dermal substitutes, an emerging add‐on to the reconstructive surgeon *armamentarium*.

## 2 CASE PRESENTATION

A 70‐year‐old retired male patient with good overall performance status was referred to the Head and Neck Surgery department of the Portuguese Institute of Oncology of Lisbon, due to a large violaceous scalp lesion with 10 months of progressive growth and associated spontaneous bleeding.

He had a personal history of cervical lymph node tuberculosis, hepatitis B virus infection, hypertension and dyslipidaemia, but no previous hospital admission record. He denied smoking or high alcohol intake. He reported a family history of unspecified cancer (brother) and leukemia (grandson).

**FIGURE 1:**
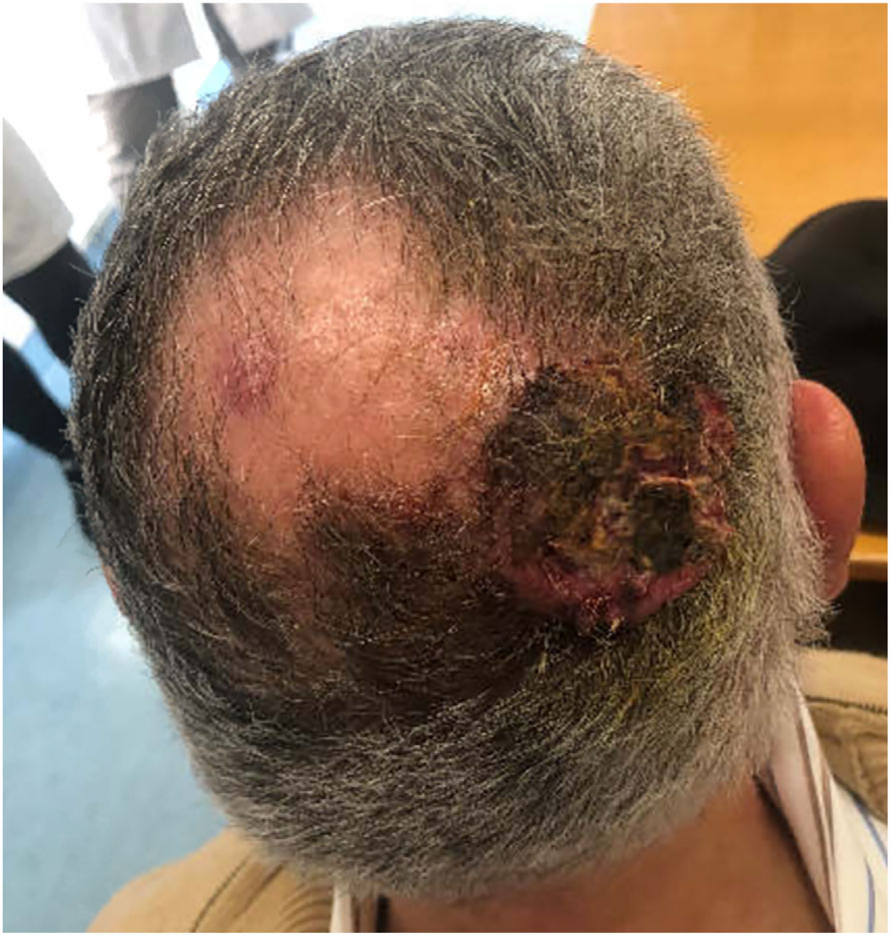
Photograph of the initial scalp skin lesion originally observed at the patient's first outpatient visit to our hospital.

On physical examination, the patient displayed a wide ulcerated red lesion of the scalp at the right occipital region, 15 × 11 cm wide, associated with cutaneous inflammation. A smaller lesion was observed close to the vertex where the patient exhibited a small area of alopecia. Also, subcutaneous lumps at left parietal and right occipital areas were palpated. Remaining neck and general examination was unremarkable (Figure 1).

At the time of referral to our institution, the patient had already undergone extensive investigations.

First, he was referred from his primary care doctor to the dermatology clinic due to unspecific erythematous and pruritic lesions in the scalp and armpit, about 3 years before definitive cancer diagnosis. According to medical records, the patient missed several follow‐up appointments, until it was proposed for local biopsy of a right occipitoparietal lesion, 3 cm wide and 1.5 cm high, reported to be infracentimetric 6 months before and clinically suspicious of a sebaceous cyst. The subsequent histologic diagnosis was AS with an epithelioid pattern, so he was referred to our institution, where revision of histological samples confirmed the diagnosis.

He was also referred with contrast‐enhanced cranioencephalic computerized tomography (CT) scan, that suggested multifocal cutaneous and subcutaneous lesions in the scalp adherent to the skull bone. No lymph node involvement or distant metastasis were identified on CT scans of neck, thorax, abdomen and pelvis.

At our hospital, cytology of the palpated subcutaneous satellite lesions confirmed AS.

The case was discussed at the multidisciplinary head and neck oncology team (MDT) and the decision was for surgical treatment.

**FIGURE 2:**
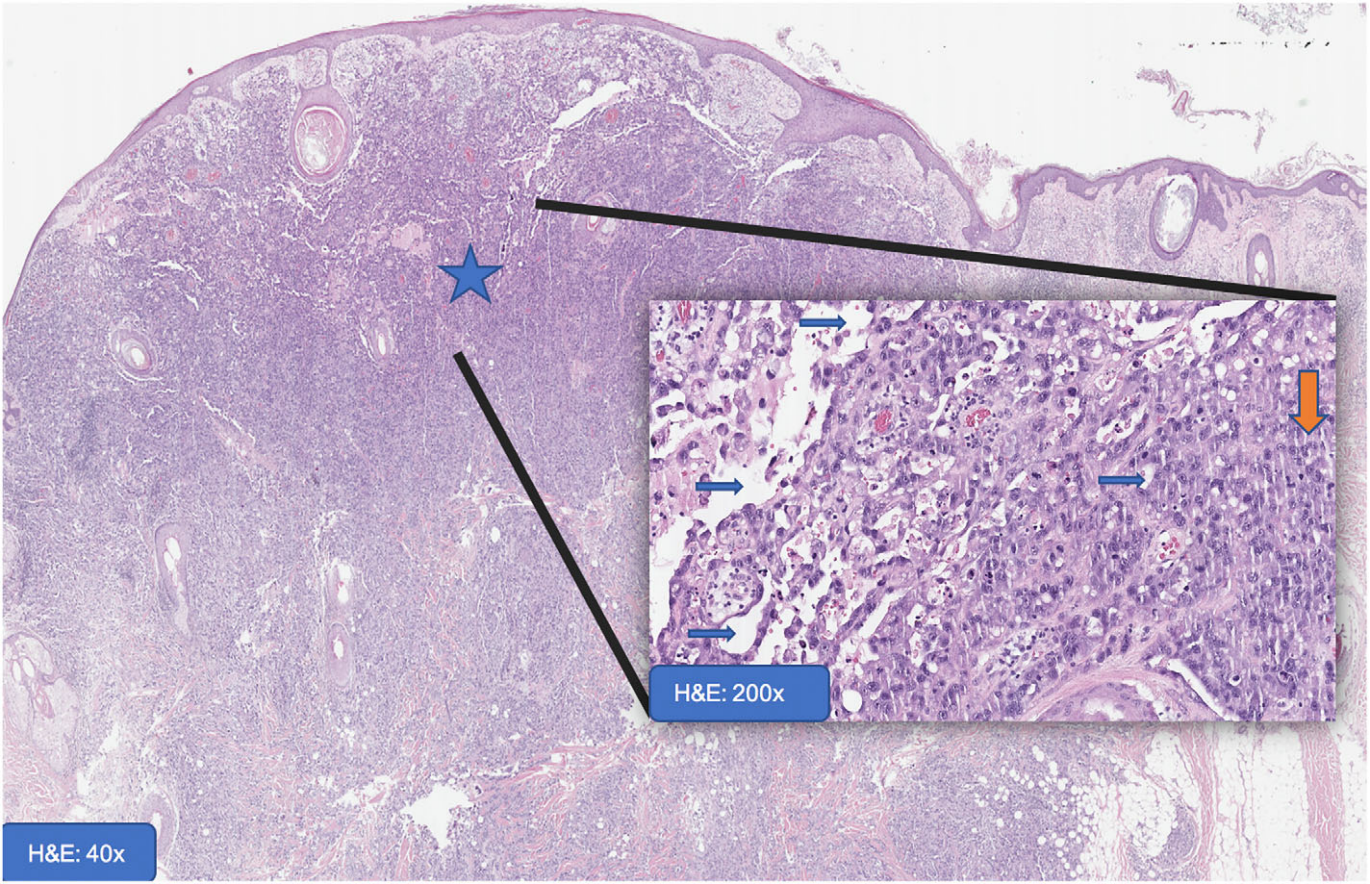
A dome‐shaped lesion (larger star) with extensive infiltrative lateral and deep growth to the subcutaneous tissue (smaller stars); luminal formation (blue arrows) indicative of vascular neoplasm and high grade atypia and poorly differentiated solid areas (larger orange arrow) were consistent  with a malignant (vascular) neoplasm diagnosis. Hematoxylin eosin stain (H&E).

An experienced team of head and neck surgeons performed the excision of the cutaneous lesions to the level of the pericranium and reconstruction of the resulting defect with galea rotation flap with full‐thickness skin graft collected from the left thigh. No complications occurred in the immediate post‐operative time and the patient was discharged after 4 days of hospital stay. The histologic result was of epithelioid AS of high degree of malignity (G3) – Fédération Nationale des Centres De Lutte Contre Le Cancer (FNCLCC) histologic grading system – with perineural spread, vascular invasion and positive deep resection margins (Figure 2).

**FIGURE 3:**
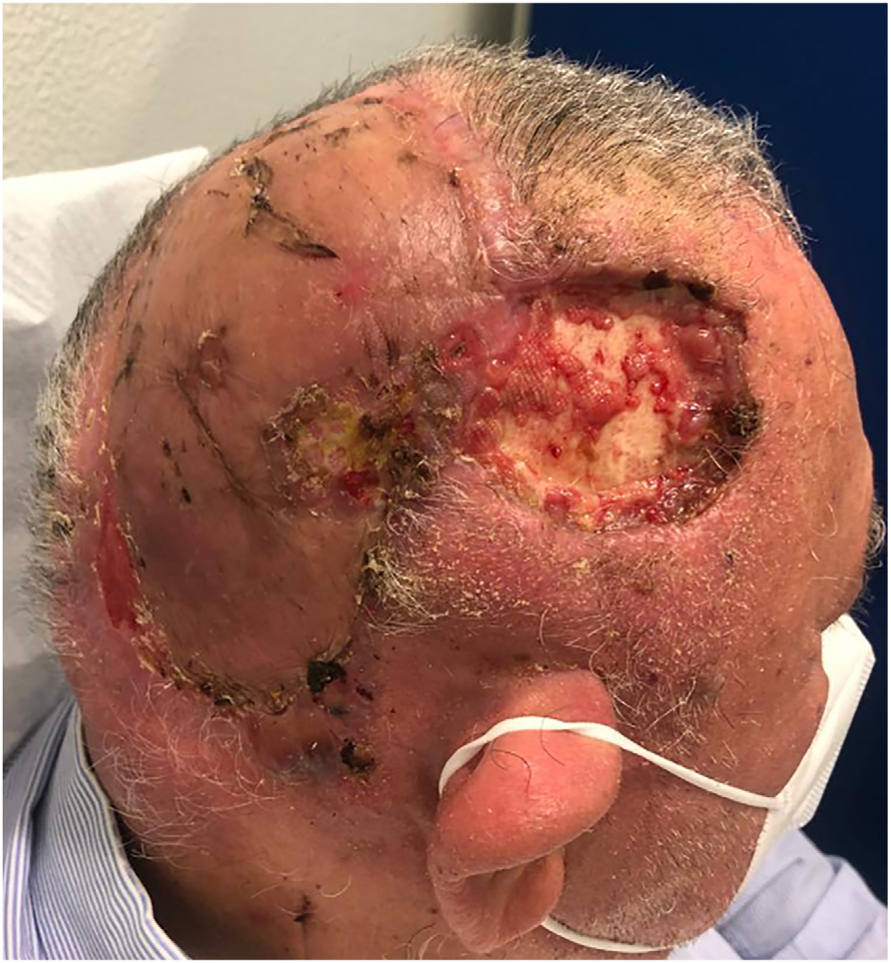
Tumor relapse around wound area, 3 weeks after first surgery.

**FIGURE 4:**
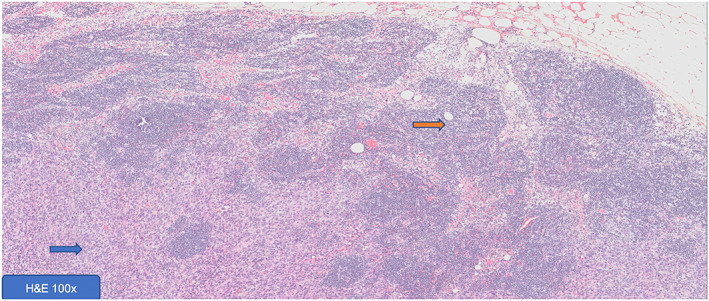
Lymph node metastasis (blue arrow) and preserved lymphoid tissue (Orange arrow). Hematoxylin eosin stain (H&E).

At 3 weeks of follow‐up, the patient presented with loss of the grafted region as well as new lesions around wound area, later confirmed to be of tumor relapse (Figure 3). Afterward, the decision of the MDT was again for surgery, which the patient accepted and was submitted about 4 weeks after the first procedure. The surgical team excised the satellite lesions *en bloc* with the grafted skin, galea aponeurosis, and outer layer of skull bone. The large scalp defect was reconstructed with heterologous acellular dermal matrices—Integra® double layer and vacuum dressing. The histologic analysis of the surgical specimen demonstrated positive deep surgical margin and microscopic lymph node tumor invasion (Figure 4).

Due to the start of COVID‐19 pandemic, the patient missed immediate clinical and wound care follow‐up after hospital stay. At 4 weeks after the second intervention, one‐third of dermal template was lost to wound infection with *Pseudomonas aeruginosa*, which resolved after 10 days of ambulatory targeted antibiotics. Moreover, the patient exhibited a de novo frontal lesion, near the anterior limit of scalp, that was later confirmed to be AS. A re‐staging positron emission tomography (PET) scan reported right occipital and left iliac crest lesions. The first one was confirmed to be a lymph node metastasis (LNM) after fine needle aspiration cytology; the second was submitted to a CT guided biopsy and was negative for neoplastic cells.

**FIGURE 5:**
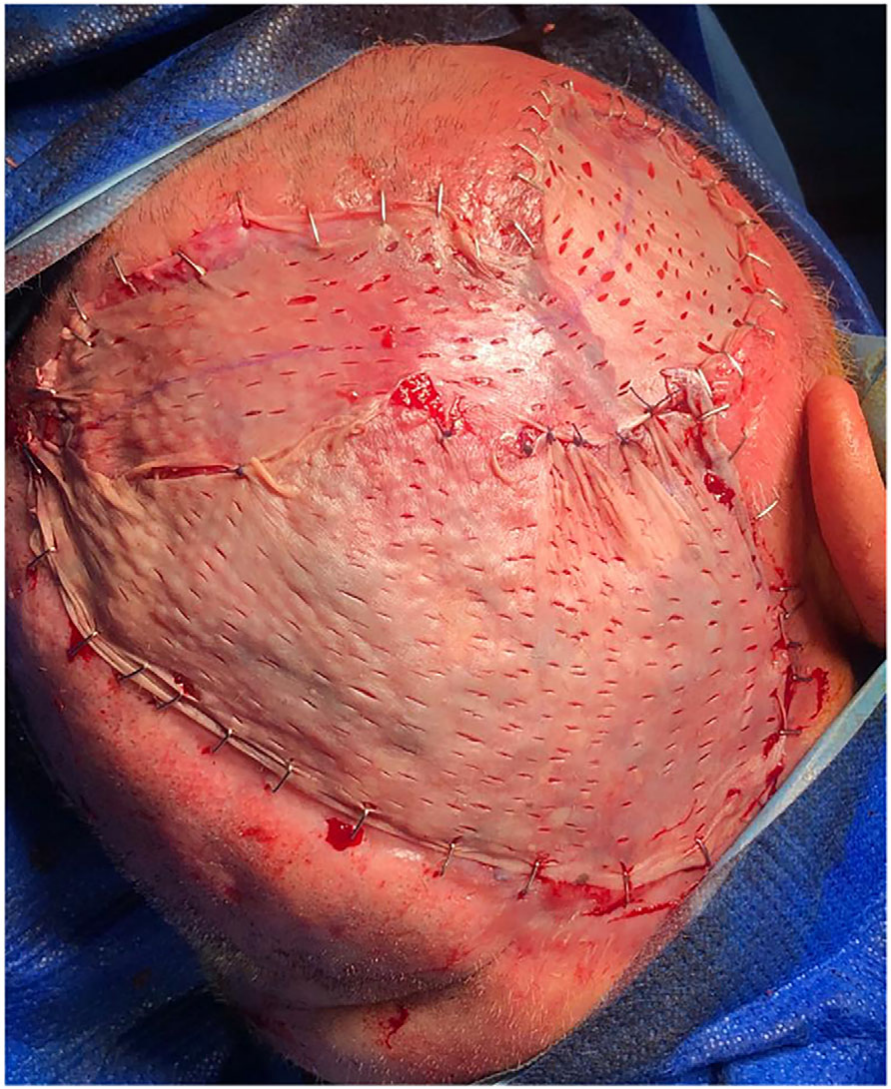
Scalp reconstruction with acellular skin substitute Integra® single layer and split‐thickness skin graft.

The case was revaluated at the multidisciplinary decision clinic, and it was decided reoperation followed by adjuvant radiochemotherapy. Thus, the patient underwent right type III modified radical neck dissection, excision of the frontal lesion with superficial ostectomy of the skull, surgical debridement of the exposed area of scalp and reconstruction of both defects with acellular skin substitute Integra® single layer and split‐thickness skin graft (Figure 5). The patient was discharged after 1 week of hospital stay with no reported medical complications. The histologic evaluation reported surgical margins free of tumor, diagnostic for AS, with associated perineural invasion, as well as 2 positive out of 19 neck lymph nodes, both at right level V and with extranodal extension.

**FIGURE 6:**
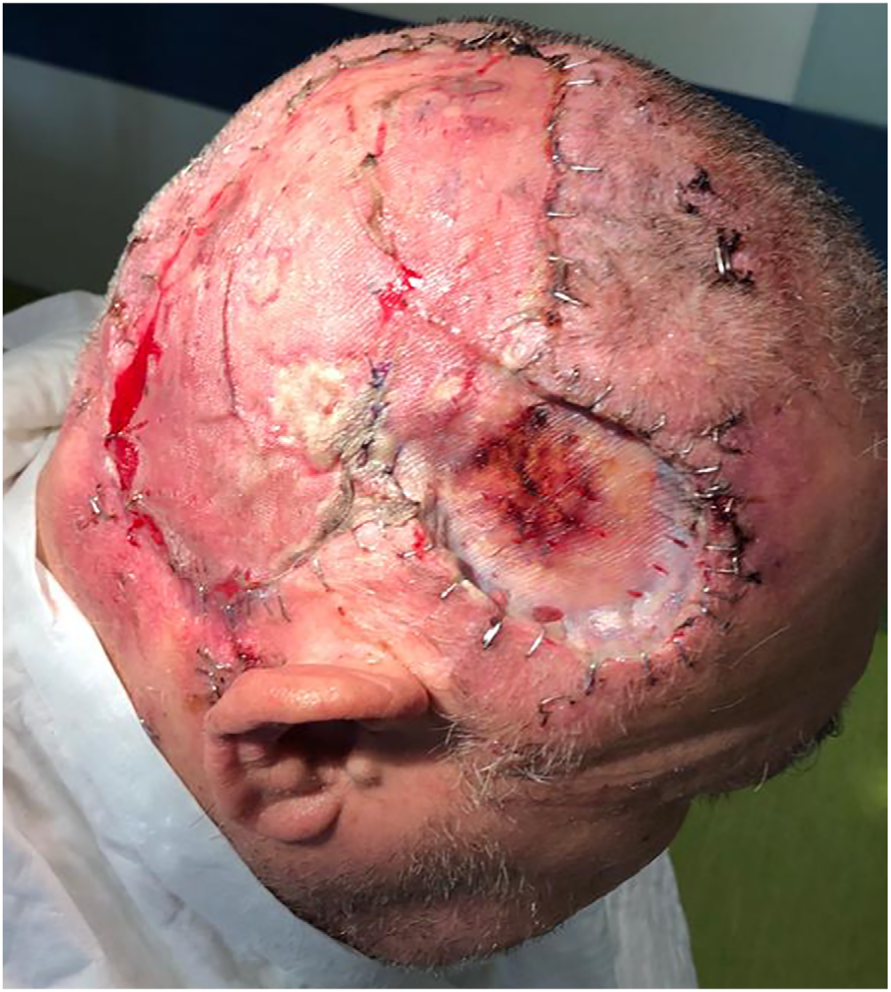
Scalp wound healing at 2 weeks follow‐up after last intervention.

At 4 weeks of follow‐up from previous intervention, the patient presented with favorable wound healing of the scalp (Figure 6). Following 1 week, the patient started adjuvant radiotherapy. He completed a total of 66 Gy in 33 sessions and 6 weeks, with reported good tolerance.

Four months after latest surgery, the patient had no symptoms, maintained good wound healing of the scalp and a clear neck physical examination after surgery. For the next 4 months the patient decided to go living far away to his countryside home because he was afraid of current pandemic situation, so that he had to be follow‐up by phone. Although he reported no suspicious symptoms or physical signs by teleconsultation, a new follow‐up PET scan reported local and regional progression of the disease, confirmed by biopsy. This time, the decision was for adjuvant chemotherapy with paclitaxel, which the patient had to delay for 2 weeks because he contracted COVID infection and needed hospitalization.

**FIGURE 7:**
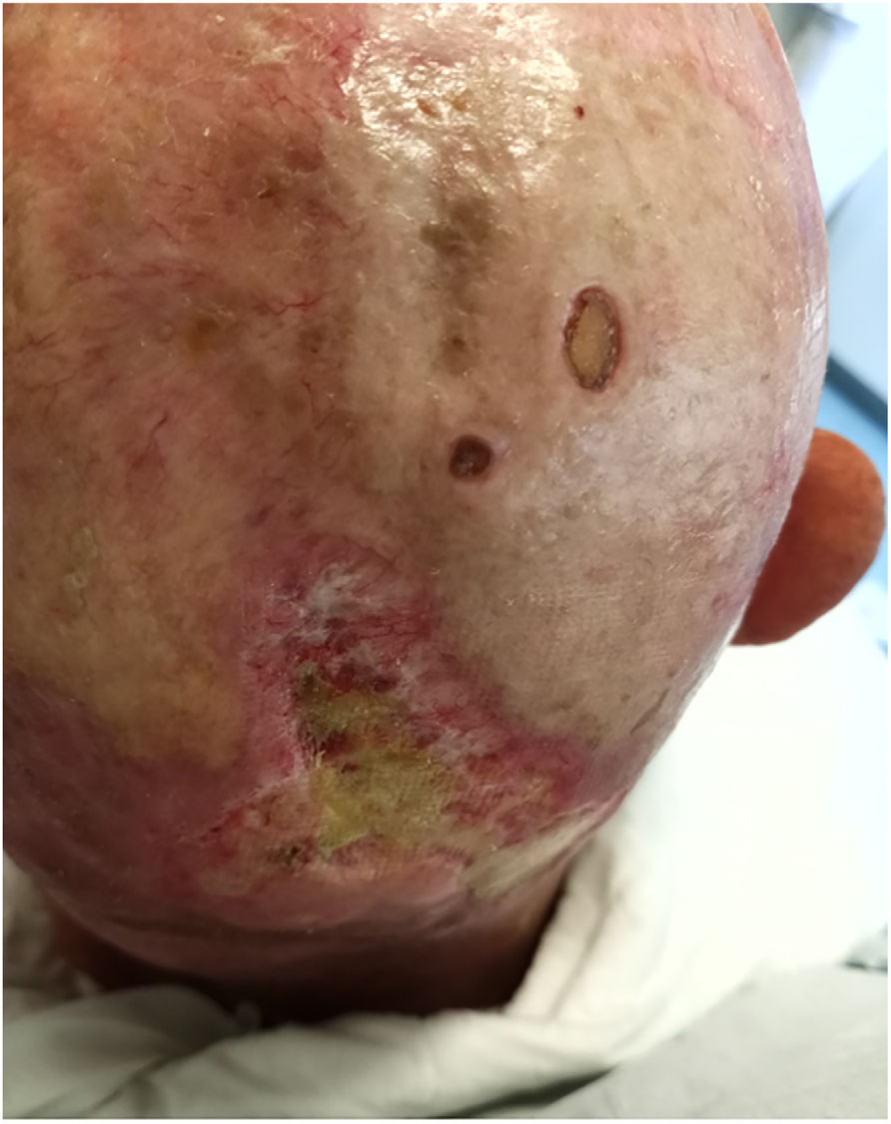
Scalp wound healing at 9 months follow‐up after last intervention.

**FIGURE 8:**
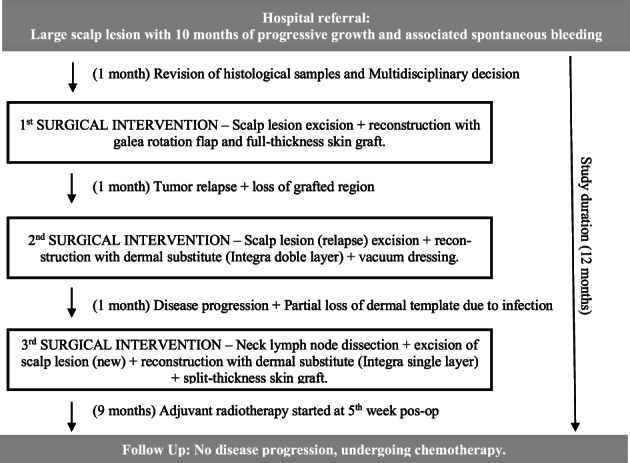
Summary outline of the treatment plan to the present case report.

Currently, at 9 months of follow‐up since last surgical procedure and about 12 months after evaluation at our hospital, the patient is undergoing chemotherapy with good tolerance, and displays no signs of further disease progression at clinical examination (Figures 7 and 8).

## 3 DISCUSSION

**FIGURE 9:**
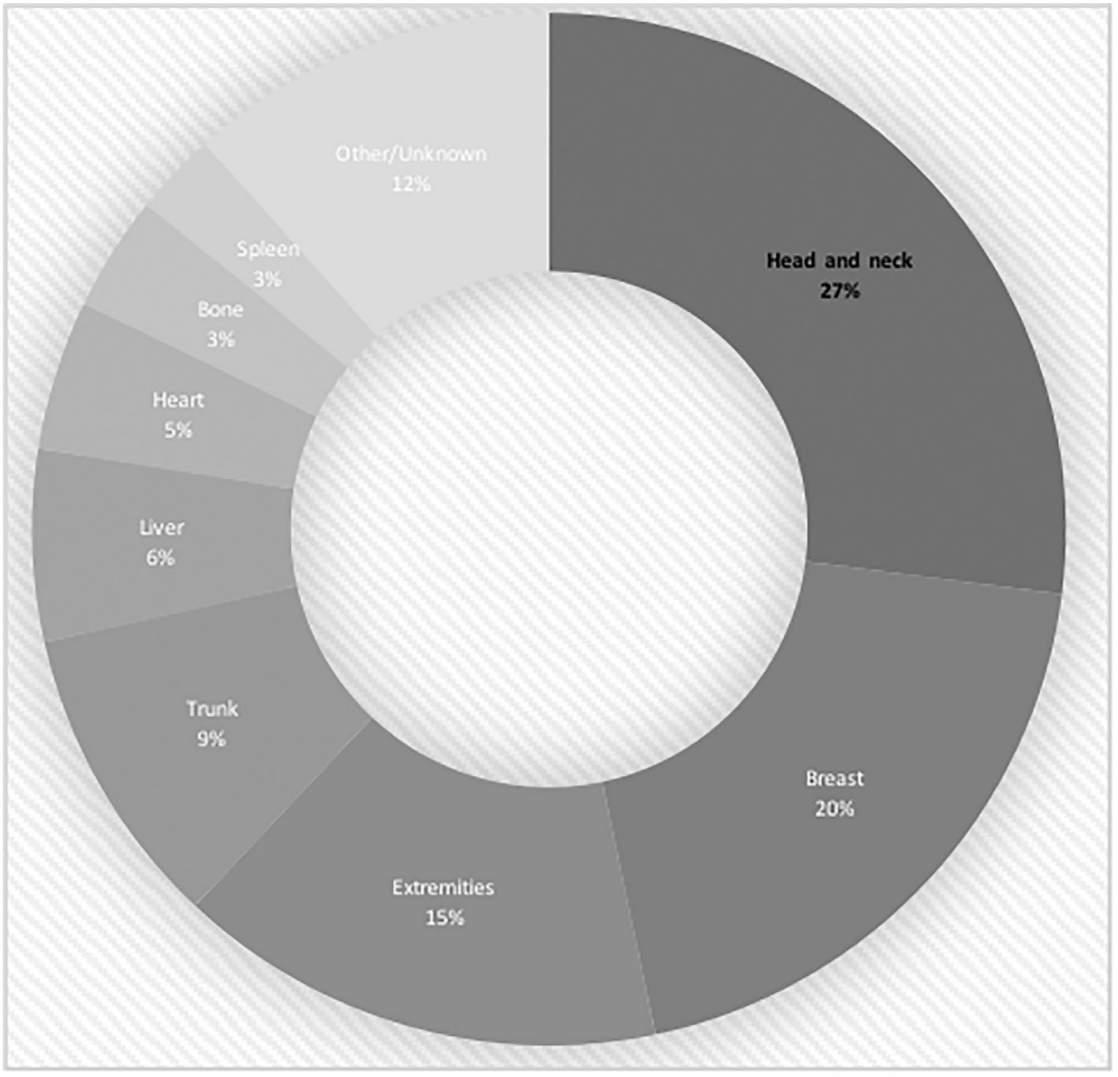
Chart with anatomical location distribution of angiosarcoma.^8^

We present a rare clinical case of a 70‐year‐old Caucasian male patient with idiopathic head and neck cutaneous AS, where the difficulty in disease control and challenging scalp reconstruction are especially evident. In order to address these issues, the surgical team resorted to a relatively recent add‐on to the reconstructive surgeon *armamentarium*—dermal substitutes—with a high degree of success. In line with what is most frequently described in the literature, the present report occurred in a male Caucasian patient, in the 60–70 years age group, located in the head and neck (Figure 9), with no determined causal factor and with clinically multifocal disease, with marked prone for superficial and deep invasion of the surrounding tissues.^5^, ^8^, ^9^, ^10^, ^11^, ^12^

The lack of initial clinical suspicion verified in this case report mirrors the challenging diagnostic nature of cutaneous AS that often presents insidiously, as a bruise‐like lesion or a purplish papule that may be mistaken for a benign lesion.^6^, ^13^ (Table 1). An adequate histological assessment is necessary, and it is usually supported by immunohistochemistry features to establish the diagnosis.^14^, ^15^, ^16^, ^17^

**TABLE 1:** Differential diagnosis of cutaneous angiosarcoma^1^ ^,^ ^2^ ^,^ ^5^

Vascular lesions	Non‐vascular lesions
Reactive and benign vascular tumors Capillary heamangiomas Juvenille Juvenille haemangioma (strawberry naevus) Cherry angioma (Campbell de Morgan spot) Pyogenic granuloma Cavernous haemangiomas Epithelioid haemangioma Vascular ectasis (naevus flammus, spider naevus) Angiomatosis Postradiation atypical vascular lesion Intermediate grade vascular tumors Kaposi's sarcoma Epithelioid haemangioendothelioma Malignant vascular tumors Angiosarcoma Tumors of perivascular cells Haemangiopericytoma (solitary fibrous tumor)	Cutaneous melanoma Skin adnexal tumors Inflammatory/immune skin reactions (e.g. Rosacea)

AS represent some of the most clinically aggressive tumors.^5^ There are certain well‐studied risk factors (Table 2), that are more common in AS of breast and extremities.^5^ The incidence is increasing due to the higher use of radiotherapy, a known risk factor, and advances in the field of histopathology diagnosis.^5^, ^10^ Mutations at the p53 gene, present in 53.8% of patients, are considered the main pathway of malignant transformation.^9^

**TABLE 2:** Risk factors of angiosarcoma.^5^

Risk factors for angiosarcoma
Radiation Chronic lymphedema (Stewart–Treves syndrome)—postsurgery/radiotherapy, Milroy's syndrome Exogenous toxins—anabolic steroids, arsenic, foreign bodies Familial syndromes—neurofibromatosis NF‐1, mutated BRCA‐1/BRCA‐2, Maffucci syndrome, Klippel–Trenaunay syndrome

The prevalence of lymph node metastasis (LNM) of all AS is reported between 6% and 13.5%.^8^ Contrary, the present case had lymph node involvement, in line with a recent study that observed a higher prevalence (52.5%) of regional LNM in scalp ASs.^8^

Distant metastasis may occur in up to 50%.^11^ The lung is the most common site followed by bone and the liver.^8^, ^11^ There is little information on the metastatic risk of head and neck cutaneous angiosarcoma (HNCAS).^1^ Tumor depth was identified as a locoregional metastasis risk factor.^1^ Gründahl et al proposed a three level locoregional metastasis risk score, based on three characteristics of the primary tumor—head and neck localization, tumor extent and American Joint Committee on Cancer (AJCC) stage (Table 3).^1^ Our case had no report of distant metastases as determined by the two PET scans performed and the biopsy directed to an iliac crest lesion that was initially suspected but later confirmed without malignancy.

**TABLE 3:** Proposed algorithm for calculation of the locoregional metastasis risk score^1^

Variable	Assessed points					Value range
	0	1	2	3	4	
Localization		Face	Capillitium and neck			1–2
Tumor extent		One region		More than one region		1,3
AJCC stage	IA	IIA	IB	IIB	III, IV	0–4
Total						2–9
Locorregional metastasis risk score		Low		2–4		Points
		Medium		5–6		
		High		7–9		

There are no stablished guidelines for the treatment of cutaneous AS of the head and neck.^9^, ^18^ Surgery plays a central rule, particularly for localized disease.^1^, ^3^, ^4^, ^19^ However, cutaneous AS is often multifocal and infiltrates tissue far more widely than is clinically apparent.^3^, ^19^ Even with negative margin resection, approximately 25%–50% of patients may face local recurrence.^19^ Because disease is frequently unresectable, radiotherapy, chemotherapy or both are frequently used as well.^3^, ^8^, ^9^, ^11^

Our case also brings awareness to the lymph node management in angiossarcomas because cervical metastasis was identified in our patient, so an elective neck dissection (END) was performed. Usually, END is not indicated because reported LNM is low.^8^ However, Kang et al. hypothesized a rule for prophylactic neck lymph node dissection, especially when surgery was considered with curative intent, based on observations that distant metastasis alone without clinical node involvement was relatively infrequent (27.5%) and regional LNM was a very strong indicator for the subsequent systemic dissemination (nearly 100%), that could be detected within 3–6 months after clinical LNM.^8^

Our case report is an example of the difficulty of local control of head and neck sarcomas (HNSA).^10^, ^19^ Although usually smaller in size than other sites, HNSA often have disproportionately greater risk of local recurrence.^15^ This is in part because of the challenge of obtaining negative surgical resection margins and in delivering adequate levels of ionizing radiation to tumors adjacent to critical anatomic structures.^17^, ^20^

Also present in this case report is the reconstructive challenge of large scalp defects, particularly when adjuvant radiotherapy was considered.^21^, ^22^, ^23^, ^24^ The surgical team adopted the principles of the reconstructive ladder—the simplest reconstruction should be used whenever possible to provide the most functional and esthetic reconstruction—that are highly important to scalp defects, also allowing for clinical surveillance of the tumor.^25^ The surgical team employed different types of skin reconstruction technics for the scalp defects throughout the various interventions. Full‐thickness skin grafts are preferred for managing defects in esthetic zone.^23^ In comparison, split‐thickness skin graft was preferred for use in association with acellular skin substitute Integra® single layer and after partial loss of skin grafting in the frontal region.^23^ Dermal regeneration templates have been shown to improve operative time and inpatient stay, no technic limitation by size, location, or radiation of the recipient bed, better tumor surveillance due to preservation of the tumor bed and good long‐term functional and esthetic result.^24^, ^25^, ^26^ After the initial unsuccessful autologous skin grafting, the surgical team decided for dermal substitutes, that could deliver on the patient's needs of a simpler intervention for a large scalp defect while allowing clinical tumor monitoring, together with the team's large experience with this kind of reconstruction.

The skull was exposed, and the dermal substitutes covered a wide area, so it was decided to associate negative pressure wound therapy (NPWT), which is thought to promote tissue granulation and decrease wound volume by debriding devitalized tissue, decreasing bacterial colonization, promoting blood flow, and removing excess serous fluid that might inhibit wound healing.^25^, ^27^ NPWT is advantageous when used for wound preparation both before application of a skin graft (‘pre‐graft’) and after (‘post‐graft’).^28^, ^29^ When used for a skin substitute it appears to speed up the process of granulation tissue formation in difficult areas (e.g., scalp injuries, etc.), likely by induction of the granulation process, stabilization of the substitute and control of exudate.^30^ In fact, it may prove to be an alternative to free microvascular tissue transfer coverage.^28^, ^29^, ^30^

Microssurgical free tissue transfer also plays an important role in scalp reconstruction because large defects with bone exposure as well as the need for adjuvant therapy are frequent.^31^ Previous studies recommended latissimus dorsi flap, anterolateral thigh flap, and radial forearm flap.^31^ In the present case, free tissue transfer was not used because it would demand a more complex surgery as well as the reduced capability for clinical surveillance for local tumor relapse.^31^

Ultimately the appropriate reconstructive approach to the scalp is a case‐by‐case decision that depends on the wound characteristics, patient factors and available technical expertise.^32^ in addition, in line with the present case, it is pointed out that new adjuncts, such as dermal substitutes or NPWT, will continue to shape stablished reconstructive paradigms, for example, the reconstructive ladder.^32^, ^33^

There is insufficient published data of an advantage in progression‐free survival or overall survival (OS) with the use of systemic chemotherapy in localized AS.^11^, ^20^ On the other hand, systemic therapy is the cornerstone of treatment in metastatic disease, and although there is no established first‐line standard treatments, the most used ones are taxane or anthracycline‐based regimens.^11^ The treatment landscape of AS is slowly changing, namely with the use of pembrolizumab (anti‐PD1 checkpoint inhibitor) for AS with high total number of mutations (≥10 mutations/megabase), or modest results reported from clinical trials with bevacizumab, an antibody against vascular endothelial growth factor, and tyrosine kinase inhibitors.^11^

A recent metanalyses of AS of the scalp and face showed a 5‐year survival rate of 33.5%.^8^ Four predisposing factors for poor prognosis were identified: advanced age (>70 years), large size (>5 cm), site of tumor (scalp in comparison with the face), and positive margin status.^8^, ^34^ All four unfavorable prognostic factors were present in our patient.

Prognosis is also influenced by AS etiology—radiation induced (secondary) AS have poorer prognosis than sporadic types—and treatment modality—patients who undergo both radical surgery and adjuvant radiotherapy have 5‐year survival of 67%, better than those who undergo either chemotherapy plus radiotherapy or surgery alone.^8^, ^9^ Patel et al. identified the use of radiation therapy, and multimodality therapy to be independent prognostic factors for improved locoregional control and recurrence‐free survival. Moreover, the use of multimodality therapy is considered the single most important factor for prognosis and one explanation for the slight increase of OS—from the reported 33.6% of older studies to 38% of more recent ones.^21^

There is a trend toward improved outcomes in HNCAS in recent years, which may stem from greater awareness toward the disease, new radiotherapy modalities, and improved reconstruction of larger defects, namely with free tissue transfer.^12^ It is important to have in mind, however, that much of the published data on these rare tumors are based on case series, which causes difficulty in interpretation of results, and sometimes contradictory results.^1^, ^5^

The SARS‐CoV‐2 pandemic interfered at least in the follow‐up of the second and last surgical interventions of our patient, the last one motivated by fear of infection and angst of potential lethal complications due to his oncologic health status. Indeed, there is evidence that the COVID‐19 pandemic, significantly impaired emotional wellbeing in patients with cancer.^35^ There was also a 2 week delay to chemotherapy start because the patient was infected with SARS‐CoV‐2, albeit without sequelae. We hypothesize our patient's prognosis may have suffered, resulting from later detection of recurrence in the aftermath of the second intervention when follow‐up was changed from outpatient evaluation to interview by phone. Studies concerning the implications of sarscov2 pandemic in the prognosis of AS patients are lacking. However, experience from a recent prospective study on head and neck squamous cell carcinoma (HNSCC) to assess the potential implications of the coronavirus pandemic on HNSCC follow‐up, did not find a delay on recurrence diagnosis resulting from the change in follow‐up practise toward telemedicine or phone on the first year after treatment of HNSCC.^36^

In conclusion, it is important to be aware of AS in the differential diagnosis of head and neck cutaneous lesions because it may mimic a benign lesion at early stages and is often associated with difficult disease control, as it was described in our patient. Surgery is the cornerstone of treatment, frequently followed by chemoradiotherapy. Skin substitutes are a potential solution with proven efficacy in the present case to the complex reconstruction of large scalp defects caused by aggressive tumors such as AS, with the potential benefit of local surveillance, usually not suitable with other options, such as free tissue transfer.

Future studies are needed to assess whether Covid‐19 pandemic implications to follow‐up routine or delayed start of chemotherapy influence prognosis in AS patients.

## AUTHOR CONTRIBUTIONS

**Henrique Messias:** Conceptualization (supporting); data curation (equal); formal analysis (equal); investigation (lead); methodology (equal); project administration (equal); resources (equal); validation (equal); visualization (equal); writing – original draft (lead); writing – review and editing (lead). **Mariluz Martins:** Conceptualization (lead); resources (equal); supervision (lead); validation (lead); visualization (lead); writing – original draft (supporting); writing – review and editing (supporting). **Carlos Zagalo:** Conceptualization (equal); data curation (equal); formal analysis (equal); supervision (equal); validation (equal); visualization (equal). **Pedro Gomes:** Investigation (equal); project administration (lead); resources (equal); supervision (equal); validation (equal); visualization (equal).

## CONFLICT OF INTEREST

The authors have stated explicitly that there are no conflicts of interest in connection with this article.

## ETHICAL STATEMENT

Ethical approval was obtained from and in accordance with the local institutional ethics committee.

## Data Availability

Data sharing is not applicable to this article as no new data were created or analyzed in this study.
